# The effects of trastuzumab on HER2-mediated cell signaling in CHO cells expressing human HER2

**DOI:** 10.1186/s12885-018-4143-x

**Published:** 2018-03-01

**Authors:** Hamid Maadi, Babak Nami, Junfeng Tong, Gina Li, Zhixiang Wang

**Affiliations:** Department of Medical Genetics, and Signal Transduction Research Group, Faculty of Medicine and Dentistry, University of Alberta, Edmonton, AB T6G 2H7 Canada

**Keywords:** HER receptors, EGFR, HER2, HER3, Trastuzumab, Dimerization, Phosphorylation, ADCC, CHO cells

## Abstract

**Background:**

Targeted therapy with trastuzumab has become a mainstay for HER2-positive breast cancer without a clear understanding of the mechanism of its action. While many mechanisms have been suggested for the action of trastuzumab, most of them are not substantiated by experimental data. It has been suggested that trastuzumab functions by inhibiting intracellular signaling initiated by HER2, however, the data are very controversial. A major issue is the different cellular background of various breast cancer cells lines used in these studies. Each breast cancer cell line has a unique expression profile of various HER receptors, which could significantly affect the effects of trastuzumab.

**Methods:**

To overcome this problem, in this research we adopted a cell model that allow us to specifically examine the effects of trastuzumab on a single HER receptor without the influence of other HER receptors. Three CHO cell lines stably expressing only human EGFR (CHO-EGFR), HER2 (CHO-K6), or HER3 (CHO-HER3) were used. Various methods including cytotoxicity assay, immunoblotting, indirect immunofluorescence, cross linking, and antibody-dependent cellular cytotoxicity (ADCC) were employed in this research.

**Results:**

We showed that trastuzumab did not bind EGFR and HER3, and thus did not affect the homodimerization and phosphorylation of EGFR and HER3. However, overexpression of HER2 in CHO cells, in the absence of other HER receptors, resulted in the homodimerization of HER2 and the phosphorylation of HER2 at all major pY residues. Trastuzumab bound to HER2 specifically and with high affinity. Trastuzumab inhibited neither the homodimerization of HER2, nor the phosphorylation of HER2 at most phosphotyrosine residues. Moreover, trastuzumab did not inhibit the phosphorylation of ERK and AKT in CHO-K6 cells, and did not inhibit the proliferation of CHO-K6 cells. However, trastuzumab induced strong ADCC in CHO-K6 cells.

**Conclusion:**

We concluded that, in the absence of other HER receptors, trastuzumab exerts its antitumor activity through the induction of ADCC, rather than the inhibition of HER2-homodimerization and phosphorylation.

## Background

The HER family of receptor tyrosine kinases (RTKs) includes EGFR/HER1/ErbB1, HER2/ErbB2, HER3/ErbB3, and HER4/ErbB4 [1, 2]. Except for HER4, the aberrant activation of HER receptor kinase activity contributes to the tumorigenesis and progression of breast cancer [3–11]. Overexpression of EGFR, HER2 and HER3 occurs in 30–40%, 20–30% and ~ 20% of breast cancer cases, respectively [4, 11–16]. Targeting HER2 has proven to be an effective therapeutic strategy for HER2-positive breast cancer [17, 18]. Since its approval by FDA in 1998, trastuzumab, an antibody against HER2, has changed the paradigm for the treatment of HER2-positive breast cancer [18, 19]. However, after the initial success, acquired resistance to trastuzumab has gradually developed, which posts a challenge that needs to be overcome [18, 20, 21].

The activation of HER receptors are induced by homo- or hetero-dimerization [2, 22, 23]. Among HER receptors, HER2 is an orphan receptor without a direct ligand and HER3 has impaired kinase activity. The heterodimerization among various HER receptors is an important mechanism to activate all HER receptors in response to ligand stimulation [2, 15, 24, 25]. The HER2 extracellular domain is always in the extended conformation and ready to be dimerized. Therefore, HER2 is the preferred heterodimeric partner for other HER receptors [2, 26–28]. Overexpression of HER2 in cancers leads to the homodimerization and the constitutive activation of HER2 [15]. Each HER receptor displays different binding affinities for various downstream signaling proteins. While EGFR and HER2 preferentially activate the Ras-ERK pathway leading to cell proliferation HER3 preferentially activates the PI3K-AKT pathway leading to cell survival [15, 29]. The heterodimerization among various HER receptors allows them to play a flexible and complex roles in cell signaling [2, 23–25, 29–39].

HER2 has been a therapeutic target for treating breast cancer due to its overexpression in 20–30% of breast cancer patients [6, 8, 11, 40]. Trastuzumab is a recombinant humanized monoclonal antibody that binds to the juxtamembrane region of HER2 [27, 41, 42]. Trastuzumab is the first HER2-targetted therapy approved by FDA for metastatic breast cancer treatment. It showed strong antitumor effects in both mouse model and HER2-positive breast cancer patients [6, 8].

While many mechanisms have been proposed for the antitumor activity of trastuzumab, including both extracellular and intracellular actions [6, 8, 43], the exact mechanisms are not known. The extracellular action is through immune-mediated response. When bound to the target cells, the Fc portion of trastuzumab will be recognized and attacked by Fc receptor on immune effector cells, principally natural-killer (NK) cells. In vitro, this process is called antibody-dependent cellular cytotoxicity (ADCC). There are solid evidence to support ADCC as a major mechanism for trastuzumab action [44–51].

On the other hand, the data regarding the intracellular mechanisms are either controversial at the beginning or challenged by the recent data [52]. Intracellular action could be through the following mechanisms: inhibition of intracellular signal transduction, stimulation of HER2 internalization and degradation, inhibition of DNA repair, inhibition of proteolytic cleavage of the HER2 extracellular domain, and inhibition of angiogenesis [6, 8, 43]. While many recent publications claim that early studies support the role of trastuzumab in inhibiting HER2 phosphorylation [6, 52, 53], many data indicate that trastuzumab either has no effect or stimulates HER2 phosphorylation [52–56]. The data regarding the effects of trastuzumab on the dimerization of HER2, activation of major signaling pathways including AKT and ERK [6, 8, 43, 57, 58], and HER2 endocytosis/downregulation [56, 59–61] are all controversial. The data regarding the role of trastuzumab on DNA repair [62], proteolytic cleavage of HER2 extracellular domain [63], and angiogenesis [64, 65] are very limited [6].

The most controversial mechanism regarding trastuzumab function is its effect on the inhibition of HER2 activation. A major reason behind this controversy is the different cellular background of various breast cancer cells lines used in those studies. Each breast cancer cell line has a unique expression profile of various HER receptors, which could significantly affect the effects of trastuzumab. To overcome this problem, in this research we adopted a cell model that allow us to specifically examine the effects of trastuzumab on a single HER receptor without the influence of other HER receptors. We aim to conclusively determine if trastuzumab specifically binds only to HER2, and blocks HER2 homodimerization and activation. To achieve our objective, we adopted a CHO cell model. Besides the parental CHO cells that do not express any detectable HER receptors, three stable CHO cell lines that stably express only a single HER receptor including EGFR (CHO-EGFR), HER2 (CHO-K6), and HER3 (CHO-ErbB3) were employed in this research. We demonstrate that overexpression of HER2 in CHO cells resulted in the homodimerization of HER2 and the phosphorylation of HER2 at all major pY residues. Trastuzumab bound to HER2 specifically and with high affinity. Trastuzumab neither inhibited the homodimerization of HER2, nor inhibited the phosphorylation of HER2 at most phosphotyrosine residues. Moreover, trastuzumab did not inhibit the phosphorylation of ERK and AKT in CHO-K6 cells, and did not inhibit the proliferation of CHO-K6 cells. However, trastuzumab induced strong ADCC in CHO cells overexpressing HER2.

## Methods

### Cell culture and treatment

All cells were cultured at 37 °C with 5% CO2 atmosphere. Chinese Hamster Ovary (CHO) cell was purchased from ATCC (ATCC® CCL-61™, Manassas, VA). CHO cell stably expressing human EGFR (CHO-EGFR) was previously generated [66]. CHO cell stably expressing HER2 (CHO-K6) [67], and HER3 (CHO-HER3) [68] were gifts from other labs. Dulbecco’s modified Eagle’s medium (DMEM) containing 10% FBS were used for cell culture. The cells were maintained at 37 °C in a 5% CO_2_ atmosphere. To maintain the selection pressure G418 (500 mg/ml) were added to the culture medium. For treatment, cells were starved overnight at DMEM containing 1% FBS and then treated in this starvation medium. EGF, trastuzumab, normal IgG, CP-714724, or vinorelbine was added at indicated concentration for indicated time periods.

### Chemicals and antibodies

CP-724714 HER2 inhibitor was purchased from Selleckchem (Houston, TX, USA). Lapatinib and isotype control human IgG were purchased from Sigma-Aldrich (St. Louis, MO, USA). Trastuzumab was purchased from Roche (Basel, Switzerland). Mouse monoclonal anti-human HER2 (9G6) and (A-2), anti-human EGFR (A-10), and anti-human HER3 (RTJ.2) antibodies were purchased from Santa Cruz Biotechnology Inc. (Dallas, TX, USA). Rabbit polyclonal anti-human phospho-HER2 Y-1005, Y-1112, Y-1127, Y-1139, Y-1196, and Y-1248 antibodies were purchased from FroggaBio (Toronto, ON, Canada). All other chemicals were purchased from Sigma-Aldrich.

### Cell proliferation assay by MTT

Cell proliferation was determined by MTT assay Vybrant MTT Cell Proliferation Assay Kit from Invitrogen (Grand Island, NY). The detailed protocol of the assay was described previously [69]. The cells were treated with various agents including EGF, trastuzumab, normal IgG, CP-714724, or vinorelbine for 24 or 48 h. Each value is the average of at least three independent experiments.

### Cell lysates and immunoblotting

The lysis of the cells and the immunoblotting were described previously [66].

### Cross-linking assay

Cross-linking assay was employed to determine the dimerization of the receptors. CHO cells were cultured to subconfluency in 60 mm dishes. Following the treatment with EGF, trastuzumab, and normal human IgG of indicated concentrations for 1 h at 37 °C, the cells were collected and suspended in 0.2~ 0.5 ml PBS. BS3 [bis(sulfosuccinimidyl)suberate] was then added to a final concentration of 1.0~ 2.5 mM. The cross-linking reaction was conducted on ice for 2 h. To stop the reaction the quench solution (1 M Tris, pH 7.5, 1:100 dilution) was added and incubated for 15 min on ice. The final concentration of the quench solution was 10 mM. Afterwards, the cells were lysed with 1% NP-40 on ice for 1 h. The dimerization was analyzed by SDS-PAGE followed by immunoblotting. A gel of 5% was run to better separate the dimers from the monomers.

### Immunofluorescence staining assay

Cells were cultured on the immunofluorescence slides 48 h before treatment starts. After treatment period, the slides were rinsed in tris-buffered saline (TBS; 6% tris, 8.8% NaCl, 85.2% dH2O, PH = 7.6) and the cells were fixed by cold methanol for 4 min. Blocking was done with incubation of slides in 1% BSA in TBS for an hour. The slides were then treated with 1 μg/ml indicated primary antibodies in TBS for 1 h. Following rinsing in TBS for three times, the slides were incubated with 1 μg/ml FITC- and/or TRITC-conjugated secondary antibody in TBS for an hour in the dark. Thereafter, the slides were washed completely in TBS and incubated in 1 μg/ml DAPI in TBS for 5 min at room temperature in the dark. The slides were observed using a DeltaVision fluorescence microscopy system (Applied Precision Inc., Mississauga, ON, Canada).

### Antibody-dependent cellular cytotoxicity (ADCC)

ADCC of trastuzumab in CHO cells expressing HER2 or EGFR was determined by using Promega ADCC Bioassay kit according to Manufacturer’s instruction. Cultured cells were plated at the density of 15,000 cells per well in complete culture medium overnight before bioassay. On the day of bioassay, the series of concentrations of trastuzumab were added to the cells, followed by addition of ADCC Bioassay Effector Cells. The E:T ratio was 5:1. After 6 h of induction at 37 °C, Bio-Glo™ Luciferase Assay Reagent was added and luminescence was determined.

## Results

### Stable CHO cell lines expressing EGFR, HER2 and HER3

**Fig. 1:**
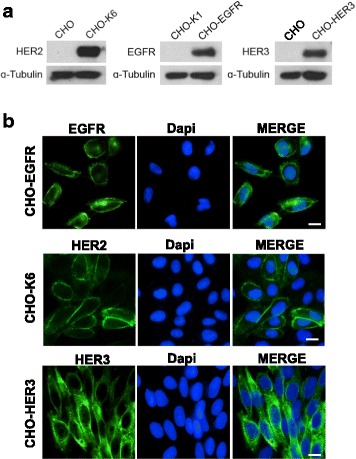
The expression of HER receptors in CHO cells stably transfected with a single HER receptor including CHO-EGFR, CHO-K6, and CHO-HER3. **a** Immunoblotting. The lysates of various CHO cells were separated by gel electrophoresis and immunoblotted with antibodies to HER receptors as indicated. The parent CHO cells (CHO) were used as control. **b** Immunofluorescence. Various CHO cells were fixed and stained with antibodies to HER receptors as indicated. The expression of HER receptor was revealed by FITC-conjugated secondary antibody (Green). Cell nuclei were counterstained with DAPI. Size bar: 10 μm

HER2 heterodimerizes with EGFR and HER3 in response to ligand stimulation [2, 15, 25]. HER2 also homodimerizes and activates in cells with over-expressed HER2 [15, 70, 71] . Most HER2-positive breast cancer cells also express either EGFR, HER3 or both, which makes it difficult to explain the observed effects of trastuzumab. Thus, to understand the effects of trastuzumab on HER2-mediated cell signaling in breast cancer cells, we plan first to study the effects of trastuzumab in CHO cells that selectively express a single HER receptor. The results from these CHO cells will unambiguously define the role of trastuzumab on HER2-mediated cell signaling under various expression profiles of HER receptors. Thus, these data could be used to accurately interpret observation in breast cancer cells. We have established CHO cell lines stably expressing EGFR (CHO-EGFR) [66]. CHO cells expressing HER2 (CHO-K6) or HER3 (CHO-HER3) were obtained from other labs [67, 68]. Parental CHO cells is used as control. We confirmed the expression of HER receptors in these cell lines by immunoblotting and immunofluorescence. As shown in Fig. 1, CHO-K6 cells expressed high level of HER2. CHO-EGFR cells expressed high level of EGFR. CHO-HER3 cells expressed high level of HER3 and the parental CHO cells did not express detectable HER2, EGFR and HER3.

### Binding of trastuzumab to HER receptors

**Fig. 2:**
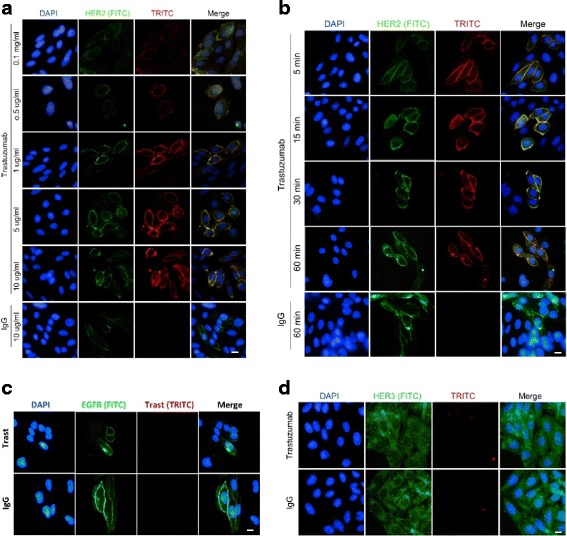
Binding of trastuzumab to HER receptors in CHO-K6, CHO-EGFR and CHO-HER3 cells as revealed by immunofluorescence. **a** and **b** The binding of trastuzumab to HER2 in CHO-K6 cells. CHO cells were treated with trastuzumab at various concentrations for 1 h (**a**) or at various time period at 10 μg/ml (**b**) as indicated. The membrane localization (binding) of trastuzumab was revealed by TRITC-conjugated donkey anti-human IgG. The localization of HER2 was revealed by rabbit anti-HER2 antibody followed by FITC-conjugated donkey anti-rabbit IgG. The cell nuclei were counter stained with DAPI. Yellow indicated the co-localization of trastuzumab and HER2. **c** The binding of trastuzumab to EGFR in CHO-EGFR cells. The membrane localization (binding) of trastuzumab was revealed by TRITC-conjugated donkey anti-human IgG. The localization of EGFR was revealed by rabbit anti-EGFR antibody followed by FITC-conjugated donkey anti-rabbit IgG. The cell nuclei were counter stained with DAPI. **d** The binding of trastuzumab to HER3 in CHO-HER3 cells. The membrane localization (binding) of trastuzumab was revealed by TRITC-conjugated donkey anti-human IgG. The localization of HER3 was revealed by rabbit anti-HER3 antibody followed by FITC-conjugated donkey anti-rabbit IgG. The cell nuclei were counter stained with DAPI. Size bar: 10 μm

While trastuzumab is an antibody to HER2, it is possible that it may weakly interact with EGFR and HER3 due the sequence homology among these receptors. Thus, we next examined the binding of trastuzumab to HER2, EGFR and HER3. We showed by immunofluorescence that trastuzumab only bound to HER2, but not EGFR and HER3 (Fig. 2). As shown in Fig. 2, at the dosage ranging from 0.1 μg/ml to 10 μg/ml, trastuzumab showed strong binding to HER2 in CHO-K6 cells. HER2 was localized to the plasma membrane (PM) in CHO-K6 cells under all conditions as expected. Trastuzumab was also located to PM, co-localizing with HER2, which indicates the binding of trastuzumab to HER2 (Fig. 2a). PM localization of trastuzumab was increased with the increased dosage. We also determined the time course of trastuzumab binding to HER2 in CHO-K6 cells. As shown in Fig. 2b, at 5 min following trastuzumab addition, trastuzumab had already been well localized to the PM, indicating a rapid binding between trastuzumab and HER2. Longer incubation only slightly increased the PM localization of trastuzumab. However, even at the high dosage of 10 μg/ml, no binding of trastuzumab to EGFR and HER3 was detectable in CHO-EGFR and CHO-HER3 cells, respectively (Fig. 2c & d). These results indicate that trastuzumab binds to HER2 specifically with high affinity.

### The effects of trastuzumab on the homodimerization of HER2

**Fig. 3:**
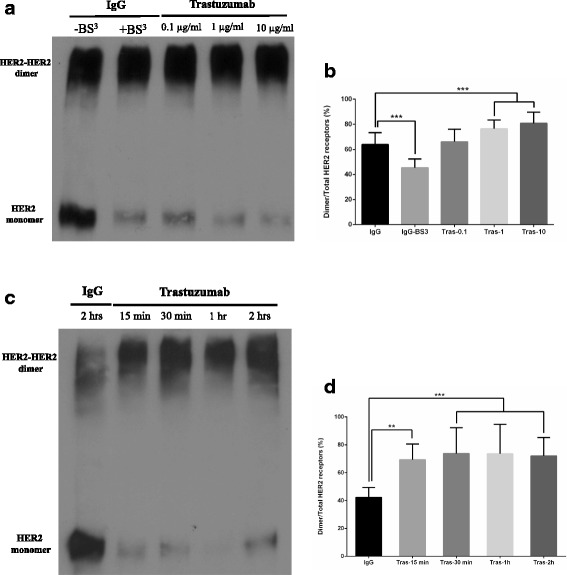
The effects of trastuzumab on HER2 homodimerization in CHO-K6 cells as revealed by crosslinking. Following trastuzumab treatment as indicated, CHO-K6 cells were treated with BS^3^ and the homodimerization of HER2 was revealed by immunoblotting as described in Materials and Methods. **a** CHO-K6 cells were treated with trastuzumab at various concentrations ranging from 0.1–10 μg/ml for 1 h. **b** Quantification of the data in (**a**). **c** CHO-K6 cells were treated with 10 μg/ml trastuzumab for various time period as indicated. Cells treated with normal human IgG were used as control. **d** Quantification of the data in (**b**). The level of HER2 homodimerization were quantitated by densitometry and expressed as the ratio of dimer/total HER2. Each value is the average of at least three experiments and the error bar is standard error. **: *p* < 0.01, ***: *p* < 0.001

So far, the reports are controversial regarding the effects of trastuzumab on the dimerization or HER2. Here we examined the effects of trastuzumab on the homodimerization of HER2 by crosslinking and immunoblotting (Fig. 3). As shown in Fig. 3, overexpression of HER2 by itself resulted in high level of HER2 homodimerization. Clearly trastuzumab did not block the homodimerization of HER2. Interestingly, with the increase of the dosage from 0.1 μg/ml to 10 μg/ml, trastuzumab slightly induced the dimerization of HER2 (Fig. 3a). The induction of homodimerization of HER2 by trastuzumab was even more visible in the time course experiments (Fig. 3b).

### The effects of trastuzumab on the phosphorylation of HER2

**Fig. 4:**
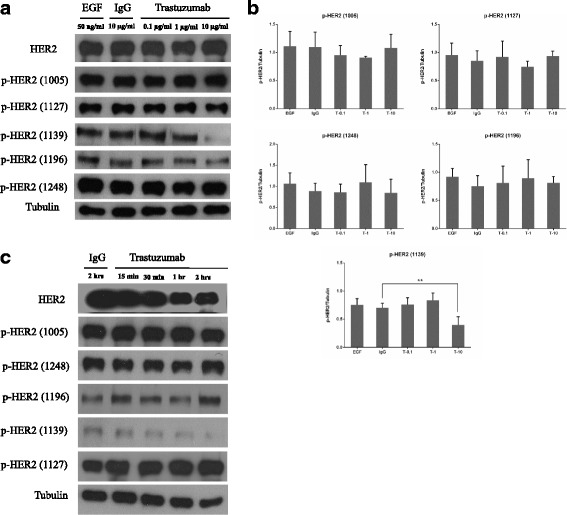
The effects of trastuzumab on HER2 phosphorylation in CHO-K6 cells by immunoblotting. **a** CHO-K6 cells were treated with trastuzumab at various concentrations for 1 h. The phosphorylation of HER2 at Y1005, Y1127, Y1139, Y1196, and Y1248 were then examined by immunoblotting as described in Materials and Methods. Cells treated with normal human IgG or EGF were used as controls. **b** Quantification of the results in (**a**). The phosphorylation level of each HER2 pY residue was normalized against the expression level of tubulin. Each value is the average of at least three experiments and the error bar is standard error. **: *p* < 0.01. **c** Time course experiments. CHO-K6 cells were treated with trastuzumab (10 μg/ml) for various time as indicated. The phosphorylation of various HER2 pY residues were examined by immunoblotting

**Fig. 5:**
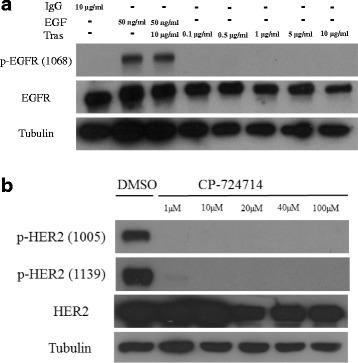
Control experiments to show the effects of trastuzumab on EGFR phosphorylation in CHO-EGFR cells and the effects of CP-724714 on HER2 phosphorylation in CHO-K6 cells. **a** The effects of trastuzumab on EGFR phosphorylation or EGF-induced EGFR phosphorylation in CHO-EGFR cells. Cells were treated with EGF and/or trastuzumab at various concentrations as indicated. The phosphorylation of EGFR was determined by immunoblotting with antibody to EGFR pY1068. **b** The effects of chemical inhibitor of HER2, CP-724714 on HER2 phosphorylation in CHO-K6 cells. Cells were treated with CP-724714 at various concentrations as indicated for 1 h. The phosphorylation of HER2 was examined by immunoblotting with antibodies against HER2 pY1005 and pY1139

**Fig. 6:**
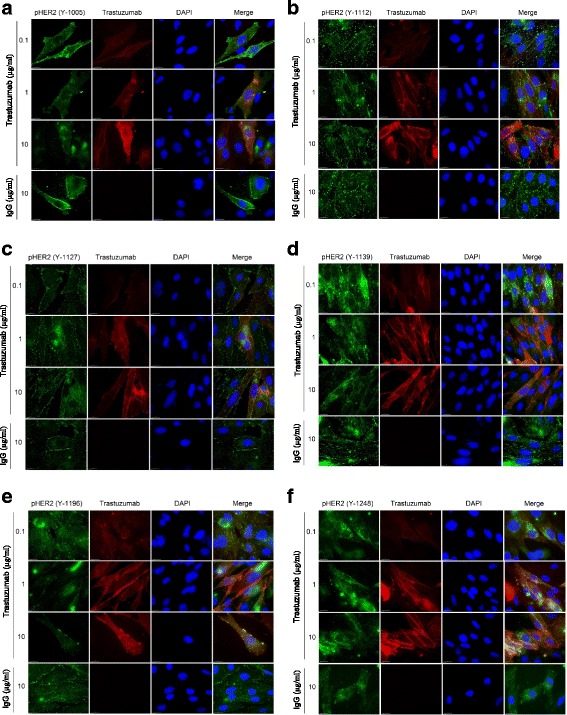
The effects of trastuzumab on HER2 phosphorylation in CHO-K6 cells by immunofluorescence. CHO-K6 cells were treated with trastuzumab at concentrations ranging from 0.1–10 μg/ml for 1 h. The phosphorylation of HER2 at Y1005 (**a**), Y1112 (**b**), Y1127 (**c**), Y1139 (**d**), Y1196 (**e**), and Y1248 (**f**) were then examined by immunofluorescence as described in Methods. The localization of trastuzumab was revealed by TRITC-conjugated donkey anti-human IgG. The localization of HER2 was revealed by rabbit anti-phosphoHER2 antibody followed by FITC-conjugated donkey anti-rabbit IgG. The cell nuclei were counter stained with DAPI. Yellow indicated the co-localization of trastuzumab and phosphoHER2. The cells treated with normal human IgG (10 μg/ml) were used as negative controls. Size bar: 10 μm

Activated HER2 phosphorylates multiple tyrosine (Y) residues at its C-terminus. We have examined the phosphorylation of following six tyrosine residues including Y1005, Y1112, Y1127, Y1139, Y1196, and Y1248 (Figs. 4, 5 and 6). As shown by immunoblotting, for the control cells treated with normal IgG, HER2 was well phosphorylated in all of the pY residues examined (Fig. 4a & b). The phosphorylation is likely due to the homodimerization induced by the overexpression of HER2. Treatment with trastuzumab at the dosage ranging from 0.1 μg/ml to 10 μg/ml did not significantly alter the phosphorylation levels of most phosphotyrosine residues including Y1005, Y1127, Y1196, and Y1248. However, trastuzumab partially inhibited the phosphorylation of Y1139. Similar to normal IgG, EGF did not have any effects on the phosphorylation of all the pY residues of HER2, which is not surprising as HER2 does not bind to EGF (Fig. 4a & b). These results were confirmed by time course experiments (Fig. 4c). Treatment from 15 min up to 2 h, did not change the phosphorylation levels of all pY residues except for pY1139 that is partially inhibited (Fig. 4c).

As controls, we have examined the effects of trastuzumab on EGFR phosphorylation in CHO-EGFR cells. As shown in Fig. 5a, EGFR was not phosphorylated in CHO-EGFR cells, and addition of EGF stimulated the phosphorylation of EGFR. Treatment with trastuzumab was not able to inhibit EGF-induced EGFR phosphorylation. Moreover, trastuzumab by itself did not have any detectable effects on EGFR phosphorylation in CHO-EGFR cells. We also examine the effects of chemical inhibitor of HER2, CP-724714 on HER2 phosphorylation in CHO-K6 cells. As shown in Fig. 5b, various concentrations of CP-724714 ranging from 1 to 100 μM significantly block the phosphorylation of HER2 at Y1005, which is in stark contrast from trastuzumab as shown in Fig. 4. Furthermore, CP-724714 also significantly inhibited the phosphorylation of HER2 pY1139, however, trastuzumab only partially inhibited pY1139. These results indicated that trastuzumab has little, if any, inhibitory effects on HER2 activation/ phosphorylation.

We further examine the effects of trastuzumab on the phosphorylation of HER2 by indirect immunofluorescence (Fig. 6). CHO-K6 cells either treated with trastuzumab or control IgG were double stained for both trastuzumab (TRITC, red) and phosphor-HER2 (FITC, green). Antibodies specific to six HER2 pY residues including Y1005, Y1112, Y1127, Y1139, Y1196, and Y1248 were used to determine the effects of trastuzumab on HER2 phosphorylation. As shown in Fig. 6, HER2 was well phosphorylated on all of these six pY residues in the absence of trastuzumab, indicating the autophosphorylation due to overexpression. Treatment with trastuzumab at the concentration ranging from 0.1 μg/ml to 10 μg/ml had no effects on the phosphorylation levels of these HER2 pY residues including Y1005, Y1112, Y1127, Y1196, and Y1248 (Fig. 6a, b, c & e). However, for pY1139, trastuzumab at 1–10 μg/ml showed some inhibitory effect (Fig. 6d).

Together, our results indicated that overexpression of HER2 resulted in strong HER2 phosphorylation in all its pY residues studied here. Addition of trastuzumab, in general, did not inhibit the phosphorylation of HER2. The only possible exception is that trastuzumab at higher dosage (1–10 ng/ml) slightly reduced the phosphorylation of HER2 at pY1139.

### The effects of trastuzumab on the activation of ERK and AKT

**Fig. 7:**
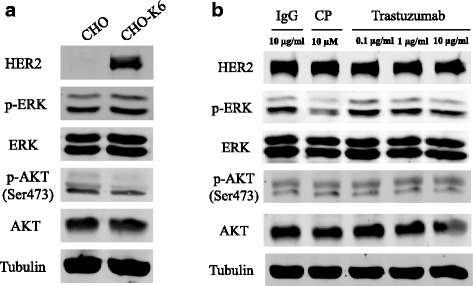
The effects of trastuzumab on the phosphorylation of ERK and AKT in CHO-K6 cells. The phosphorylation of ERK and AKT was revealed by immunoblotting as described in Materials and Methods. **a** The phosphorylation of ERK and AKT in CHO parental cells and in CHO-K6 cells. **b** The effects of trastuzumab on the phosphorylation of ERK and AKT in CHO-K6 cells. Cells were treated with trastuzumab with indicated concentrations for 7 h. Cells treated with normal human IgG was used as negative control and cells treated with CP-714724 (CP) was used as positive control

We finally examined the activation of ERK and AKT. The ERK and AKT activation was measured by their phosphorylation. As shown in Fig. 7a, the ERK phosphorylation level is higher in CHO-K6 cells than the control CHO cells, which suggests that overexpression of HER2 increased ERK activation. However, we did not observe the increase in AKT phosphorylation, which is not surprising as HER2 homodimer has very limited effects on the activation of PI3K-AKT pathway. We next examined the effects of trastuzumab on the phosphorylation of ERK and AKT in CHO-K6 cells. As shown in Fig. 7b, treatment with trastuzumab did not block the phosphorylation of ERK and AKT.

### Trastuzumab-induced ADCC

**Fig. 8:**
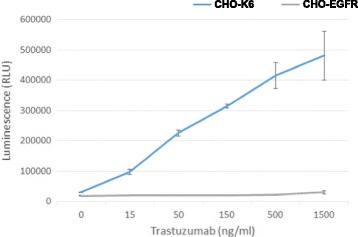
Trastuzumab-induced ADCC in CHO-K6 and CHO-EGFR cells. Trastuzumab-induced ADCC was examined in both CHO-K6 and CHO-EGFR cells by using Promega ADCC Bioassay kit as described in Methods

Above results suggests that trastuzumab did not inhibit HER2 dimerization and phosphorylation. Thus, it is interesting to find out if trastuzumab can induce ADCC in cells overexpressing HER2. Trastuzumab-induced ADCC in CHO-K6 and CHO-EGFR cells was determined by using Promega ADCC Bioassay kit. As shown in Fig. 8, trastuzumab induced very strong ADCC in CHO-K6 cells, but not in CHO-EGFR cells.

### The effects of trastuzumab on the proliferation of CHO-K6 cells

We next determined if trastuzumab inhibits the proliferation of cells with overexpressed HER2. MTT cell proliferation kit was used to assess the proliferation of various CHO cells including CHO parental cells, CHO-EGFR, CHO-K6, and CHO-HER3 cells. Non-treated Cells were used as negative controls, and the cells treated with vinorelbine (an anticancer drug) were used as positive controls.

**Fig. 9:**
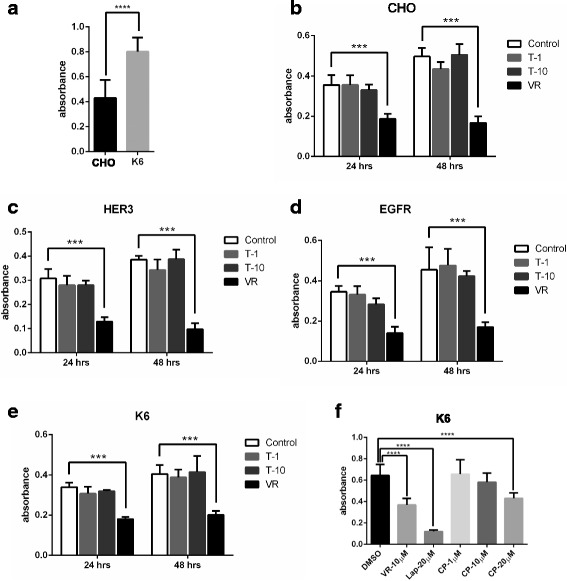
The effects of trastuzumab on the proliferation of CHO, CHO-EGFR, CHO-K6, and CHO-HER3 cells. The cell proliferation was examined by MTT assay as described in Materials and Methods. **a** The effects of HER2 overexpression on the proliferation of CHO cells. Cell proliferation of both CHO parental cells and CHO-K6 cells was examined. **b**-**e** The effects of trastuzumab on the proliferation of CHO, CHO-EGFR, CHO-K6 and CHO-HER3 cells. Cell were treated with various concentration of trastuzumab as indicated for 24 and 48 h. Non treated cells were used as negative control and the cells treated with vinorelbine (VR) were used as positive controls. **b** CHO cells. **c** CHO-HER3 cells. **d** CHO-EGFR cells. **e** CHO-K6 cells. **f** The effects of other HER2 inhibitors on the proliferation of CHO-K6 cells. CHO-K6 cells were treated with HER2 kinase inhibitors lapatinib (Lap) and CP714724 of indicated concentration. Each value is the average of at least three experiments and the error bar is standard error. ***: *p* < 0.001. ****: *p* < 0.0001

We first determined if overexpression of HER2 in CHO cells stimulates cell proliferation by comparing CHO-K6 cells with the parental CHO cells. As shown in Fig. 9a, the proliferation rate of CHO-K6 cells is much higher than that of CHO cells, which indicates that overexpression of HER2 stimulates cell proliferation. We next examined the effects of trastuzumab on cell proliferation. It is not surprising that treatment with trastuzumab for either 24 or 48 h had no effects on the proliferation of CHO, CHO-EGFR and CHO-HER3 cells as these cell did not express HER2 (Fig. 9b-d). Interestingly, even for CHO-K6 cells that overexpressed HER2, trastuzumab at high dosage did not have any effect on their proliferation (Fig. 9e). However, vinorelbine significantly inhibited the proliferation of all these CHO cells following 24 or 48 h incubation (Fig. 9b-e). Moreover, HER2 kinase inhibitors including Lapatinib and CP714724 significantly inhibited the proliferation of CHO-K6 cells when at high dosage (Fig. 9f). Our data indicated that trastuzumab did not inhibited the proliferation of CHO-K6 cells that overexpressed HER2.

## Discussion

The most controversial mechanism regarding trastuzumab function is its effect on the inhibition of HER2 activation. A major reason behind this controversy is the different cellular background of various breast cancer cells lines used in those studies. Each breast cancer cell line has a unique expression profile of various HER receptors, which could significantly affect the effects of trastuzumab due to the heterodimerization among HER receptors. In this research we adopted a CHO cell model. Besides the parental CHO cells that do not express any detectable HER receptors, three stable CHO cell lines that stably express only a single HER receptor including EGFR (CHO-EGFR), HER2 (CHO-K6), and HER3 (CHO-ErbB3) were employed in this research. Our cell model system avoided the interference of other HER receptors, and is very suitable to study the effects of trastuzumab on the homodimerization of HER2 and the phosphorylation of HER2 homodimers. We aim to conclusively determine if trastuzumab specifically binds only to HER2, and blocks HER2 homodimerization and activation.

We showed that trastuzumab only bound to HER2 specifically and with high affinity. Trastuzumab did not bind to EGFR and HER3 even at high dosage (10 ng/ml) (Fig. 2). Most HER2-positive breast cancer cells also express EGFR and HER3, our finding suggest that any trastuzumab effects on these cells must be initiated through the interaction between trastuzumab and HER2.

We next examined the effects of trastuzumab on HER2 dimerization. HER2 is an orphan receptor and does not have a ligand. However, HER2 are heterodimerized with EGFR in response to EGF stimulation and heterodimerized with HER3 in response to HRG [1]. HER2 is also homodimerized when overexpressed in cells. CHO-K6 cells only expresses a single HER receptor HER2, not EGFR, HER3 or HER4. Thus our results are regarding the effects of trastuzumab on the homodimerization of HER2.

We showed that in CHO-K6 cells HER2 was mostly dimerized, likely due to the overexpression (Fig. 3). This is not surprising. As revealed by crystal structures of the HER2 extracellular region, HER2 adopts an extended configuration, which resembles the configuration of EGFR seen in each molecule of an EGFR dimer. Thus, ErbB2 possesses a constitutive, or ligand independent, activated conformation, which allows the HER2 homodimerization when overexpressed [1, 27, 72].

We also showed that trastuzumab did not block the homodimerization of HER2 (Fig. 3). While it is originally proposed that trastuzumab acts to block HER2 dimerization, so far, no research has been done to determine the effects of trastuzumab on the homodimerization of HER2. Given the fact that trastuzumab binds to the juxtamembrane region of HER2 [27], which is not essential for HER2 dimerization, our results are not surprising. What surprising is that our data suggest that trastuzumab at high dosage actually enhanced the homodimerization of HER2 (Fig. 3). While we are not certain how trastuzumab stimulates the homodimerization of HER2, it is possible that it functions through HER2 transmembrane domain. Many data support the role of HER2 transmembrane domain in HER2 dimerization and activation [27]. Parts of juxtamembrane region has also been implicated in HER2 dimerization and activation [73–75]. As trastuzumab binds to the extracellular juxtamembrane region of HER2, it will likely affect the function of HER2 transmembrane domain and juxtamembrane region in terms of HER2 dimerization. It is possible that somehow the specific effects of trastuzumab enhanced the interaction between two HER2 transmembrane domains and thus increased HER2 homodimerization as we observed here.

It has been believed that trastuzumab functions to inhibit HER2 activation/phosphorylation and HER2-mediated cell signaling [6, 52, 53]. However, our data indicated that trastuzumab only had very limited effects on HER2 phosphorylation. Among six pY residues examined in this research, HER2 had no effects on the phosphorylation of pY1005, pY1112, pY1027, pY1196, and pY1248 (Figs. 5 and 6). While HER2 decreased the phosphorylation of pY1139, which is a much weaker inhibition when compared with CP-724714 (Figs. 5, 6 and 7). In general, this is consistent with our observation regarding the role of trastuzumab in HER2 dimerization. Trastuzumab did not block HER2 dimerization, thus it did not block HER2 phosphorylation. It is not clear how the effects of HER2 transmembrane domain on HER2 dimerization affect the phosphorylation of HER2. Some research indicated the presence of an alternative dimerization mode of HER2. In this mode, HER2 dimerization is mediated by both transmembrane domain and the cytoplasmic juxtamembrane region of HER2. Such a dimerization mode exert inhibiting effects on the HER2 kinase activity [73–75]. Thus, in theory, the enhanced dimerization through the interaction of transmembrane domain and the juxtamembrane region could result in the inhibition of certain HER2 phosphorylation including pY1139. Recently, some researches with various breast cancer cell lines have shown that trastuzumab did not significantly alter HER2 phosphorylation [53–56, 76]. Moreover, there is one research shows the enhanced phosphorylation of pY1248 in response to trastuzumab [52].

Our results suggest that trastuzumab has, if any, limited effects on HER2-mediated intracellular signaling. Indeed, when we examined the effects of trastuzumab on the phosphorylation of ERK and AKT, we showed that trastuzumab did not block the phosphorylation of both ERK and AKT in CHO-K6 cells (Fig. 7). Together, our data indicate that trastuzumab did not significantly alter HER2 activation and HER2 mediated intracellular signaling in the absence of other HER receptors. However, we need to be cautious to apply these findings to breast cancer cells. CHO cell is derived from hamster ovary, thus the expressed human HER2 may not be coupled well with downstream signaling cascades.

We then examined if trastuzumab induces ADCC in CHO-K6 cell. We showed that trastuzumab indeed induces strong ADCC in CHO-K6 cells (Fig. 8). This is specifically due to the expression of HER2 in CHO-K6 cells as there is no ADCC observed in CHO-EGFR cells (Fig. 8). The role of trastuzumab in the induction of ADCC in HER2-positive breast cancer cells have been consistently well supported by many researches [44–51]. Our results confirmed the role of trastuzumab in the induction of ADCC in a simple but specific cell setting.

We also showed that trastuzumab did not affect cell proliferation in CHO-K6 cells (Fig. 9). Some reports indicated that trastuzumab had little effect on proliferation and survival [58, 77]. However, other reports indicated that trastuzumab inhibited ErbB2 activation, and decreased the activation of ERK and PI3K-AKT pathways, which leads to reduced cell proliferation [57]. Given that trastuzumab has little effects on the phosphorylation of HER2, it is likely that trastuzumab has no effects on HER2-mediated cell signaling leading to cell proliferation. Although trastuzumab induces ADCC in CHO-K6 cell, under the culture conditions used for the MTT assay, no effector cells were present and no ADCC response are expected. It is interesting to note that our above finding are different from the observation by Ghosh [78]. Ghosh et al. reported that trastuzumab inhibited HER2 homodimer-mediated ERK phosphorylation and cell growth. The difference could be due to the different model system used in these two studies. The HER2 receptor used in the research by Ghosh et al. is fused with FKBP, and the receptor homodimerization is induced by a chemical linker AP1510 that dimerizes the receptor intracellularly through the fused FKBP.

It should be noted that while we observed strong inhibition of HER2 phosphorylation by CP724714 at 1 μM (Fig. 5b). We only observed the inhibition of CHO-K6 cell proliferation at much higher CP724714 concentration (Fig. 9f). We are not sure what cause this discrepancy, however, there are several possible explanations. Firstly, at 1 μM, CP724714 may not completely inhibit Her2 phosphorylation, we can see weak phosphorylation of Y1139 in Fig. 5b. There could be weak phosphorylation of HER2 at other pY residues that were not examined. A weak HER2 phosphorylation may still be sufficient to support cell growth. Secondly, there could be the existence of kinase-independent effects of HER2 receptors. There are many reports supporting the existence of kinase-independent cell signaling of various receptor tyrosine kinases including EGFR and Insulin receptor [79–82].

## Conclusion

Together, in this research we adopted a cell model that allow us to specifically examine the effects of trastuzumab on a single HER receptor without the influence of other HER receptors. Three CHO cell lines stably expressing only human EGFR, HER2, or HER3 were used. These model system allow us to specifically examine the effects of trastuzumab on the homodimerization of HER2 and the phosphorylation of HER2 homodimers. We demonstrate that overexpression of HER2 in CHO cells results in the homodimerization of HER2 and the phosphorylation of HER2 at all major pY residues. Trastuzumab binds to HER2 specifically and with high affinity. Trastuzumab does not inhibit the homodimerization of HER2. Trastuzumab does not inhibit the phosphorylation of HER2 at most phosphotyrosine residues.

We also observed that trastuzumab neither inhibits the phosphorylation of the downstream signaling proteins including ERK and AKT, nor inhibits the proliferation of CHO cells overexpressing HER2. However, caution is needed to apply these findings to breast cancer cells. However, trastuzumab induces strong ADCC in CHO cell overexpressing HER2. We concluded that trastuzumab exerts its antitumor activity through the induction of ADCC, rather than the inhibition of HER2-mediated cell signaling in the absence of other HER receptors.

## Abbreviations

ADCC: Antibody-dependent cellular cytotoxicity
CHO cells: Chinese Hamster Ovary cells
EGF: Epidermal growth factor
EGFR: Epidermal growth factor receptor
ERK: Extracellular signal-regulated kinases
FDA: Food and Drug Administration
HER2: Human epidermal growth factor receptor 2
HER3: Human epidermal growth factor receptor 3
HER4: Human epidermal growth factor receptor 4
MTT: (3-(4,5-dimethylthiazol-2yl)-2,5-diphenyltetrazolium bromide
NK cells: Natural-killer cells
PM: The plasma membrane
RTK: Receptor tyrosine kinase
